# *Toxoplasma gondii* infection in domestic cats and northern fur seals of the Pribilof Islands, Alaska

**DOI:** 10.1016/j.ijppaw.2025.101096

**Published:** 2025-06-11

**Authors:** Tania Dawant, Richard Gerhold, Danielle Scott, Chunlei Su, Lauren Divine, Mike Williams, Rolf Ream, Tom Gelatt, Colleen Duncan

**Affiliations:** a College of Veterinary Medicine, University of Tennessee, Knoxville, TN, USA; b College of Veterinary Medicine and Biomedical Sciences, Colorado State University, Fort Collins, CO, USA; c Ecosystem Conservation Office, Aleut Community of St. Paul Island, St. Paul, AK, USA; d NOAA Fisheries, Protected Resources/Alaska Region, Anchorage, AK, USA; e NOAA Fisheries, Alaska Fisheries Science Center, Marine Mammal Lab, Seattle, WA, USA

**Keywords:** *Toxoplasma gondii*, Laaqudan, Northern Fur seal, *Callorhinus ursinus*, Domestic cats, Pribilof Islands

## Abstract

Disseminated toxoplasmosis has been described in northern fur seals, however little is known about the significance and epidemiology of this pathogen in free ranging populations. We tested archived serum from a subset of domestic cats (*Felis catus*) and adult northern fur seals (*Callorhinus ursinus*; NFS*)* in the Pribilof Islands, Alaska, for *T. gondii* antibodies and feline fecal samples for *T. gondii* oocysts. Of the 37 cats, two (5.4 %) were seropositive and all (n = 36) fecal float samples were negative. Of the 225 NFS serum samples, 37 (16.4 %) were positive by modified agglutination tests. There was no statistically significant difference in the seroprevalence of NFS by year or region of animal capture. These findings suggest that *T. gondii* exposure in both cats and NFS is present but appears lower in this region compared to other studied areas.

## Highlights

• 16 % of northern fur seals from the Pribilof Islands had *T. gondii* antibodies.
• 5 % of cats on St. Paul Island were seropositive but no *T. gondii* oocysts were found in feces.
• T. gondii exposure appears lower in this region than in other areas.

## Graphical abstract

**Figure:**
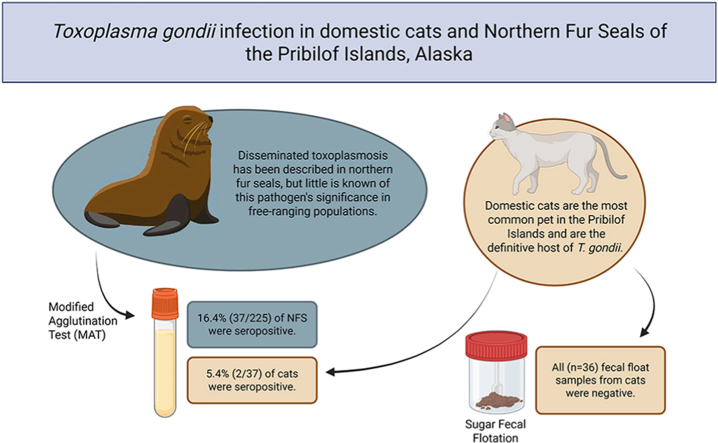

*Toxoplasma gondii* is an apicomplexan protozoan parasite that can infect many warm-blooded animals, including humans, other mammals, and birds (Dubey, 2010). Though historically considered to have a terrestrial life cycle, *T. gondii* has been identified in a range of marine mammals (Dubey et al., 2020). These infections are indicative of the contamination of marine waters with oocysts from wild or domestic felids, the only definitive hosts of the parasite. For some marine species, such as Sea otters (*Enhydra nereis*) in southern California (Miller et al., 2004) and Hawaiian monk seals (*Monachus schauinslandi) (*Barbieri et al., 2016*)*, the pathogen can cause significant morbidity and mortality as a result of multi-organ infections.

The Pribilof Islands in the Bering Sea are home to the world's largest breeding stock of northern fur seals (*Callorhinus ursinus;* NFS or laaqudan in Unangam Tunuu, first language of St. Paul Unanga $\hat{x}$ or Aleut Peoples). NFS are a highly migratory species ranging across the North Pacific Ocean (Fig. 1), and the Pribilof Islands population segment is currently estimated to be around 450,000 (Young et al., 2024). This population was intentionally culled in the 1950s and 1960s by the US federal government, declined for unknown reasons through the 1970s (York and Hartley, 1981), appeared stable in the 1980s and 1990s and has been declining steadily since 1998 (Towell et al., 2006). The species is currently listed as vulnerable by the IUCN.

**Fig. 1:**
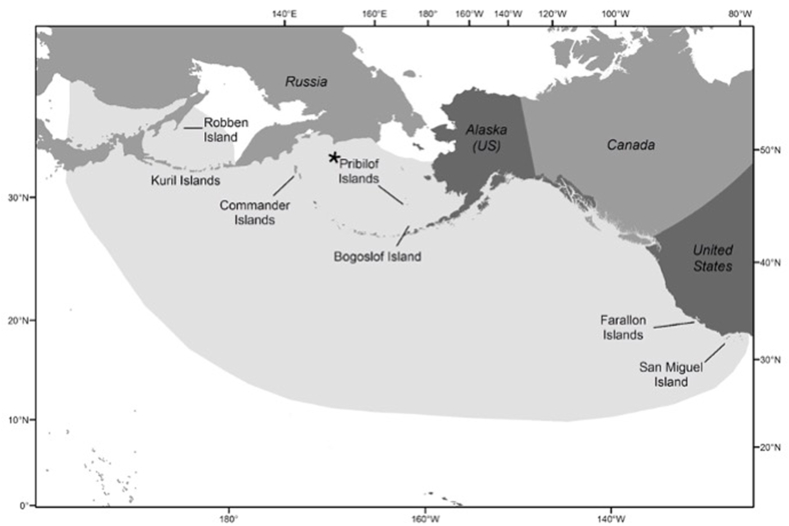
Map of winter range and breeding sites for northern fur seals (Musbach et al., 2024). Samples used in this study were collected on the Pribilof Islands (designated by **∗**) of St. Paul and St. George.

The cause of the recent NFS decline remains unidentified, and the role of infectious disease is poorly understood. Knowledge of *T. gondii* in NFS is particularly limited; a NFS literature review from 1972 to 2021 identified only one relevant publication (Cortés et al., 2022). That report documented severe, disseminated pathology with intralesional *T. gondii* tachyzoites in a single NFS found stranded in California (Holshuh et al., 1985). Domestic cats (*Felis catus*) are the most common companion animals in the two Pribilof Island communities, St. Paul (SNP) and St. George (STG); dogs have been prohibited since the 1970s to prevent negative impacts on NFS. According to the 2020 US Census, the populations of SNP and STG are 413 and 67, respectively with estimated cat populations of 40 on SNP and 3 on STG. Our objective was to investigate the prevalence of *T. gondii* in the domestic cats and NFS of the Pribilof Islands, Alaska.

Serum (n = 37) and fecal (n = 36) samples from cats were collected on SNP during mobile veterinary clinics in October 2022 and 2023. The fecal samples were collected opportunistically from litter boxes. Serum from NFS on SNP and STG was collected by the National Oceanic and Atmospheric Administration on seven occasions between 2006 and 2023 as part of routine monitoring. Modified agglutination tests (MAT) for *T. gondii* antibodies were performed on the previously frozen sera in the University of Tennessee Microbiology Laboratory as previously described (Desmonts and Remington, 1980), starting at a 1:25 dilution. Fecal flotation with centrifugation was performed on fresh fecal samples using Sheather's sugar solution method as part of the veterinary clinic services.

Two of the 37 cats (5.4 %) were positive for *T. gondii* antibodies. The titers were both 1:50 and both positive cats were from multi-cat households with no other positive housemates. All fecal samples were negative for *T. gondii* oocysts. Two hundred and twenty-five NFS serum samples were available for MAT testing. Samples were collected from adult female NFS at 4 locations on SNP (Polovina Cliffs, Reef, Vostochni, Zapadni Reef) and South rookery on STG in 7 different years (2006, 2007, 2009, 2012, 2013, 2022, 2023). Of all samples, 37 (16.4 %) had *T. gondii* titers ≥1:25 (Table 1). There was no statistically significant difference in the frequency of infected NFS across the locations (X-squared = 1.8911, df = 4, p-value = 0.7558) or years (X-squared = 10.964, df = 6, p-value = 0.08949).

**Table 1:** Distribution of NFS *T. gondii* MAT titers.

	Negative	1:25	1:50	1:100	1:200	1:800	1:1600	1:3200	Total
Count	188	12	7	9	4	1	3	1	225

These results expand our understanding of *T. gondii* in the Pribilof Islands and marine ecosystems more broadly. The seroprevalence of cats on SNP is notably lower than a meta-analysis of 31 studies estimating a pooled prevalence of 35 % (95 % CI 28–43) for domestic cats in North America (Montazeri et al., 2020). The prevalence of *T. gondii* in domestic cats varies with age, sex and feeding behavior and there can be significant variability even within cities (Dabritz and Conrad, 2010). The source of exposure for the two seropositive cats is unknown and it is possible that they were exposed prior to their arrival on the island. Failure to identify oocysts in feces is not unexpected given that only 1 % of infected cats are shedding oocysts at any time (Elmore et al., 2010).

NFS exposure to *T gondii* was more common than cats in this region. Relative to a synthesis of *T. gondii* infections in marine mammals from 2009 to 2020, the seroprevalence of NFS remains lower than that of other marine species, such as sea otters, in which clinical disease has been widely reported (Dubey et al., 2020). It is also lower than species that share a geographic range with NFS, like the California sea lions (*Zalophus californianus*, 32.9 % IFA >1:40) sampled in California (Carlson-Bremer et al., 2015). Such differences may reflect relative exposure opportunities for marine species based on time spent in water with significant oocyst contamination such as coastal California where feral cats have been identified as the source of the heavy environmental parasite burden (Zhu et al., 2023). Different serological tests used may also affect seroprevalence (Macrì et al., 2009), particularly in wildlife where validation studies can be difficult to conduct.

Although *T. gondii* exposure appears relatively uncommon in both domestic cats and NFS on the Pribilof Islands, its confirmed presence underscores the need for awareness and continued monitoring. Given the parasite's potential for zoonotic transmission through both environmental contamination and the consumption of tissue cysts in raw or undercooked meat (Tenter et al., 2000; Dabritz and Conrad, 2010), public health resources to investigate unusual findings in subsistence foods may be warranted as NFS is a critically important subsistence food for the Pribilof Island communities. While the seroprevalence observed in NFS on the Pribilof Islands, AK, is lower than in some other marine mammal species, the source of their environmental exposure remains unclear. Should clinical toxoplasmosis be detected on the Pribilof Islands, ensuring the parasite is genotyped will be a critical to understanding exposure sources. Future research should focus on determining the specific *T. gondii* genotypes present in the region, as pathogenicity can vary significantly (Shwab et al., 2014; Shapiro et al., 2019). Genetic analysis can also be used to track many different trends, including the geographic origins of the genotype (Shwab et al., 2018). Finally, expanded efforts to detect and characterize oocysts shed by local felids, alongside continued serological surveillance in NFS, would provide valuable insights into transmission dynamics.

## CRediT authorship contribution statement

**Tania Dawant:** Writing – review & editing, Writing – original draft, Validation, Methodology, Investigation, Formal analysis, Conceptualization. **Richard Gerhold:** Writing – review & editing, Writing – original draft, Supervision, Methodology, Investigation, Conceptualization. **Danielle Scott:** Writing – review & editing, Writing – original draft, Project administration, Investigation, Conceptualization. **Chunlei Su:** Writing – review & editing, Writing – original draft, Supervision, Methodology, Investigation, Formal analysis. **Lauren Divine:** Writing – review & editing, Writing – original draft, Investigation, Funding acquisition, Conceptualization. **Mike Williams:** Writing – review & editing, Writing – original draft, Investigation, Funding acquisition, Conceptualization. **Rolf Ream:** Writing – review & editing, Writing – original draft, Resources, Investigation, Conceptualization. **Tom Gelatt:** Writing – review & editing, Writing – original draft, Supervision, Investigation, Conceptualization. **Colleen Duncan:** Writing – review & editing, Writing – original draft, Supervision, Project administration, Methodology, Investigation, Funding acquisition.

## CRediT authorship contribution statement

**Tania Dawant:** Writing – review & editing, Writing – original draft, Validation, Methodology, Investigation, Formal analysis, Conceptualization. **Richard Gerhold:** Writing – review & editing, Writing – original draft, Supervision, Methodology, Investigation, Conceptualization. **Danielle Scott:** Writing – review & editing, Writing – original draft, Project administration, Investigation, Conceptualization. **Chunlei Su:** Writing – review & editing, Writing – original draft, Supervision, Methodology, Investigation, Formal analysis. **Lauren Divine:** Writing – review & editing, Writing – original draft, Investigation, Funding acquisition, Conceptualization. **Mike Williams:** Writing – review & editing, Writing – original draft, Investigation, Funding acquisition, Conceptualization. **Rolf Ream:** Writing – review & editing, Writing – original draft, Resources, Investigation, Conceptualization. **Tom Gelatt:** Writing – review & editing, Writing – original draft, Supervision, Investigation, Conceptualization. **Colleen Duncan:** Writing – review & editing, Writing – original draft, Supervision, Project administration, Methodology, Investigation, Funding acquisition.

## Conflicts of interest

The authors have no conflicts of interest to declare.
